# Poisoning by Morphine Successfully Treated with Strychnia

**Published:** 1887-01

**Authors:** W. M. Barry

**Affiliations:** Abilene, Texas


### CORRESPONDENCE.

### POISONING BY MORPHINE—SUCCESSFULLY TREATED WITH STRYCHNIA.

*Letter From Dr. W. M. Barry, Abilene.*

EDITOR DANIEL'S TEXAS MEDICAL JOURNAL:

AT your request I submit a brief report of the case of morphine poisoning, of which I made mention in my last. It occurred in April, (1886). I neglected to take notes at the time—understand I claim no priority in this treatment. Having failed under the old line of treatment with Belladonna, (atropia) and having read the report of two or three cases successfully treated with nux-vomica, by Dr. A. C. Davidson of Sharon, Georgia, and nux vomica or strychnia being a more rational antidote, I made up my mind to give it a trial on the first opportunity. As it happened the first case presenting itself was a fine one for experiment.

Case—Old man A. aged 69. A drunkard for forty years, had been on a debauch for eight or ten days; and his domestic relations being very unhappy, concluded he would end his career by morphine. As best ascertained he took about four gra. of sul. morphia. When first discovered, he was in a profoundly comatose state with contracted pupils, stertorous breathing, &c. In fact a typical case of opium narcosis. I was called at once. I asked Dr. L. A. Grizzard to go with me, and we found him in the above condition. We at once used ¼ gr. apomorphia hypodermically, also used a stomach pump, but all to no effect. By this time we were compelled to produce artificial respiration to prevent death. Dr. G. agreeing to the use of strychnia, remained with the case while I went to the drug store and had this ℞ put up. Strych. sul. gr. j. Acetic Acid. z j. Agua. Dist. z j. Of this we used 30 dr every twenty minutes, giving Strych Sul. gr. ¼ hypodermically every twenty minutes till the one gr. was given, without any appreciable effect. I had the prescription duplicated, doubling the *dose* of the *strychnia* which gave ½ gr. to the dose. In about twenty minutes after the first administration of this last, the old man had a tetanic convulsion which lasted probably one-half, to one minute—not hard. After this passed off he relaxed, the surface became warm, and he began breathing without aid; and in, I suppose, an hour's time was able to answer questions, and begged that we cease the flaggellation; and in three hours was joking about the case, declaring his intention of accomplishing his purpose, that he was sorry they found us, next time he would hide, &c. He still lives.

